# Evaluation of Synthetic 2,4-Disubstituted-benzo[*g*]quinoxaline Derivatives as Potential Anticancer Agents

**DOI:** 10.3390/ph14090853

**Published:** 2021-08-26

**Authors:** Islam Zaki, Sara A. Abu El-ata, Eman Fayad, Ola A. Abu Ali, Ali H. Abu Almaaty, Ahmed S. Saad

**Affiliations:** 1 Pharmaceutical Organic Chemistry Department, Faculty of Pharmacy, Port Said University, Port Said 42526, Egypt; 2 Chemistry Department, Faculty of Science, Port Said University, Port Said 42526, Egypt; sara_adel174@yahoo.com; 3 Biotechnology Department, Faculty of Sciences, Taif University, P.O. Box 11099, Taif 21944, Saudi Arabia; e.esmail@tu.edu.sa; 4 Chemistry Department, College of Science, Taif University, P.O. Box 11099, Taif 21944, Saudi Arabia; O.abuali@tu.edu.sa; 5 Zoology Department, Faculty of Science, Port Said University, Port Said 42526, Egypt; ali_zoology_2010@yahoo.com; 6 Pharmacology and Toxicology Department, Faculty of Pharmacy, Port Said University, Port Said 42526, Egypt; mosa1200@yahoo.com

**Keywords:** quinoxaline, naphthalene, cytotoxicity, MCF-7, cell cycle analysis, Bax, Bcl2, docking

## Abstract

A new series of 2,4-disubstituted benzo[*g*]quinoxaline molecules have been synthesized, using naphthalene-2,3-diamine and 1,4-dibromonaphthalene-2,3-diamine as the key starting materials. The structures of the new compounds were confirmed by spectral data along with elemental microanalyses. The cytotoxic activity of all synthesized benzo[*g*]quinoxaline derivatives was assessed in vitro against the breast MCF-7 cancer cell line. The tested molecules revealed good cytotoxicity toward the breast MCF-7 cancer cell line, especially compound **3**. The results of topoisomerase IIβ inhibition assay revealed that compound **3** exhibits potent inhibitory activity in submicromolar concentration. Additionally, compound **3** was found to cause pre-G1 apoptosis, and slightly increase the cell population at G1 and S phases of the cell cycle profile in MCF-7 cells. Finally, compound **3** induces apoptosis via Bax activation and downregulation of Bcl2, as revealed by ELISA assay.

## 1. Introduction

Cancer is one of the most challenging problems worldwide [1]. Breast cancer exceeds lung cancer in terms of mortality rate, with more than two million patients every year (more than 6.9% mortality rate) [2,3]. Targeted therapy has become an important type of cancer treatment, as it could reduce the activity of a specific target or prevent binding to a specific receptor [4,5]. A lot of efforts of pharmaceutical companies in the development of anticancer drugs have focused on targeted therapy for the treatment of different types of cancer [6,7]. In addition, due to the rapid progress in understanding the molecular pathways involved in cell cycle regulation, there is a motivation to target different steps during cell cycle, in order to control cancer cell proliferation and to avoid drawbacks observed with traditional chemotherapeutic agents [8,9,10]. Furthermore, the pro-apoptotic protein Bax and the ant-apoptotic protein Bcl2 are signs of apoptosis induction, which may be useful targets in the mitochondrial-dependent pathway of apoptosis [11,12]. Therefore, apoptosis induction in cancer cells can be used as a new weapon against cancer cell proliferation [13,14].

**Figure 1:**
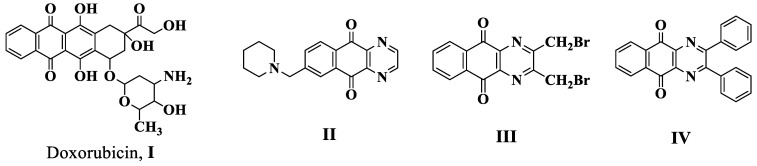
Structure of doxorubicin **I** and benzo[*g*]quinoxaline derivatives **II–IV**.

Benzo[*g*]quinoxalines are a promising class of potent anticancer agents that display broad-spectrum in vivo and in vitro activity against different types of tumor cell lines [15,16]. They are considered the chromophore-modified analogs of mitoxantrone, a synthetic analogue of Doxorubicin (Dox) **I** [17]. In addition, compounds having three to four rings give the optimal intercalation with human DNA, leading to reading errors during the replication step in the cell cycle [18,19]. For example, Benzo[*g*]quinoxaline derivatives **II** exhibited cytotoxicity comparable to that of Dox against the HCT-15 cancer cell line [20]. Furthermore, benzoquinoxalinedione **III** exhibited cytotoxicity and was highly active, especially against sarcoma [21]. 1,4-diazaanthraquinone **IV** was proven to induce apoptosis of breast cancer, which activates p53 and triggers apoptosis of tumor cells, as shown in Figure 1 [22].

Based on the above facts, and as a continuation of our search for new anticancer agents [23,24,25,26], new benzo[*g*]quinoxaline molecules were synthesized. All the synthesized molecules were evaluated for their in vitro cytotoxic activity against the breast MCF-7 cancer cell line. Additionally, the most active molecule was further evaluated for topoisomerase inhibition and for its apoptotic inducing activity.

## 2. Results and Discussion

### 2.1. Chemistry

**Scheme 1:**
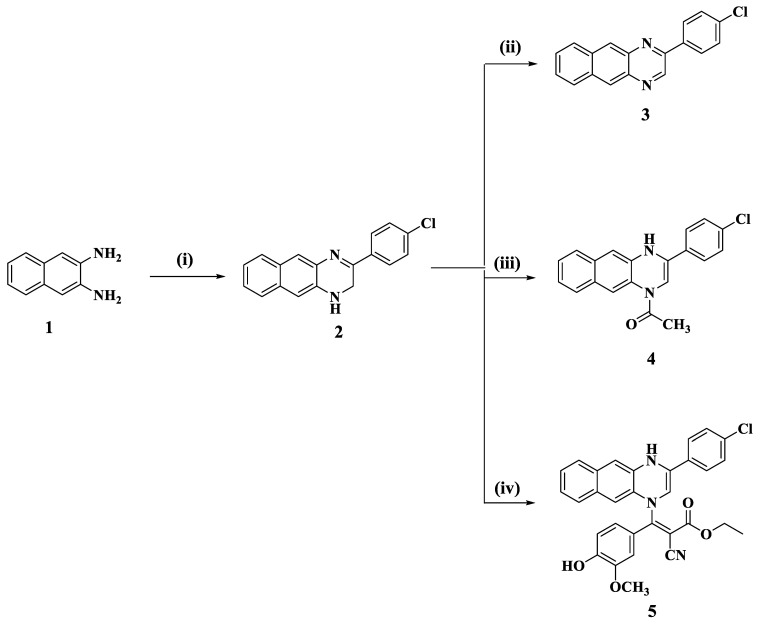
Synthesis of 2-(4-chlorophenyl)benzo[*g*]quinoxaline. Reagents and reaction conditions: (**i**) 4-chlorophenacyl bromide, AcONa/MeOH, (**ii**) Acetic anhydride/reflux, (**iii**) Acetic anhydride/CH_3_CN, reflux, (**iv**) ethyl β-aryl-α-cyanoacrylate/reflux.

Here, we synthesized 3-(4-chlorophenyl)-1,2-dihydrobenzo[g]quinoxaline (**2**) using naphthalene-2,3-diamine (**1**) as the key starting material via the condensation of compound **1** with 4-chlorophenacyl bromide in methanol in the presence of fused sodium acetate. Dehydrogenation of compound **2** in refluxing acetic anhydride led to the formation of 2-(4-Chlorophenyl)benzo[*g*]quinoxaline (**3**) [27], (Scheme 1).

**Figure 2:**
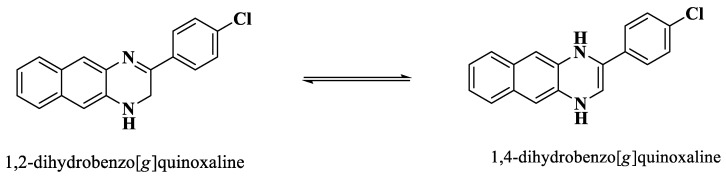
Two isomers of compound **2**.

The structure of the compounds **2** and **3** were confirmed by the spectral data. In the IR spectrum of compound **2** a band in the region 3225 cm^−1^ characteristic of the NH group was observed, while in the IR spectrum of compound **3**, the band of NH group was absent. From study, the ^1^H-NMR spectrum of compound **2** showed the structure of this compound in two isomers, as shown in Figure 2.

Proton signals of NH and CH_2_ groups in 3,4-dihydrobenzo[*g*]quinoxaline isomers were observed at δ 8.19 and 4.19 ppm, respectively, while the proton signals of two NH groups in 1,4-dihydrobenzo[*g*]quinoxaline were observed at δ 9.58 and 9.01 ppm as singlet signals and the proton of olefinic NH-CH= appeared at δ 7.34 ppm as singlet signal.

Additionally, the ^13^C-NMR spectrum of compound **2** showed the presence of five characteristic carbon signals at δ 151.02, 150.61, 150.50, 148.01, and 145.33 ppm due to the C=N, =CN groups. In addition, the ^13^C-NMR spectrum of compound **2** also showed carbon signals at δ 37.72 and 37.33 ppm assigned to carbon atom of methylene group (CH_2_).

The ^1^H-NMR spectrum of compound **3** showed two sharp singlet signals at δ 9.66 and 8.80 ppm due to the proton of azomethene (CH=N) groups and two protons of H5 and H10 of benzo[*g*]quinoxaline **3**. The residue protons of benzo[*g*]quinoxaline (**3**) appeared as multiplet signals in the region at δ 7.68–8.46 ppm due to protons of 4-chlorophenyl and benzene ring. The ^13^C-NMR spectrum of compound **3** showed two signals at δ 150.49 and 145.34 ppm due to the carbons of C=N groups. In addition, the ^13^C-NMR spectrum of compound **3** displayed 14 signals in the region at δ 138.32–127.53 ppm due to the carbons of naphthalene and 4-chlorophenyl rings.

**Scheme 2:**
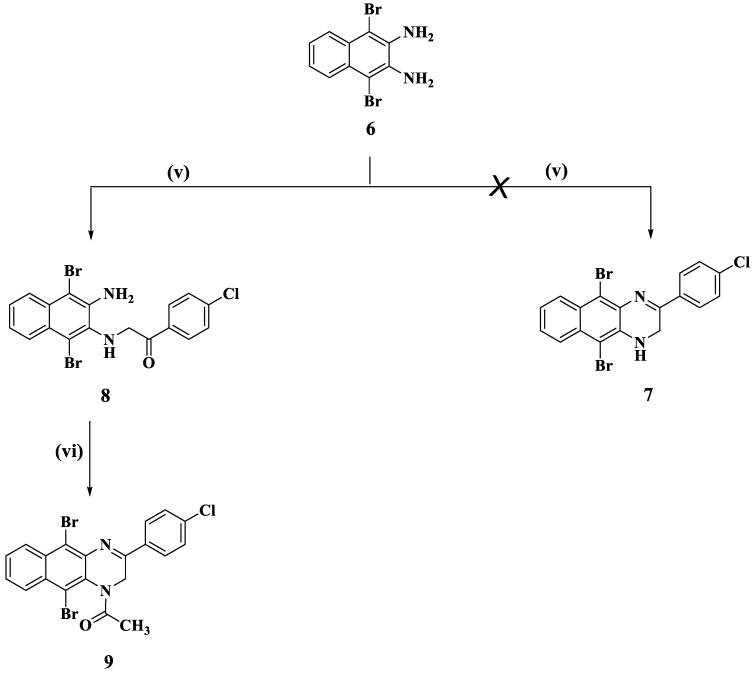
Synthesis of compounds **8** and **9**. Reagents and reaction conditions: (**v**) 4-chlorophenacyl bromide, AcONa/MeOH, and (**vi**) piperidine/AcOH.

In addition, reaction of 2-(4-chlorophenyl)-3,4-dihydro-benzo[*g*]quinoxaline (**2**) with acetic anhydride in the presence of acetonitrile afforded 2-(4-chlorophenyl)-4-acetyl-3,4-dihydro-benzo[*g*]quinoxaline (**4**). The ^1^H-NMR spectrum of compound **4** showed the structure of this compound in two isomers, as shown in Scheme 2. The proton signal of NH group in 4-acetyl-2-(4-chlorophenyl)-1*H*-benzo [g]quinoxaline (**4**) appeared at δ 9.32 as singlet signal, while the olefinic proton of this isomer was observed at δ 7.35 with aromatic protons as multiplet signal. The ^13^C-NMR spectrum of compound **4** supported the formation of two isomers of these compounds, as it showed that the presented three carbon signals at δ 150.55, 44.48, and 23.79 ppm due to the C=N, -CH_2_N, and methyl groups of the isomer 4-acetyl-2-(4-chlorophenyl)-3,4-dihydro-benzo[*g*]quinoxaline, respectively, while the ^13^C-NMR spectrum displayed carbon signals at δ 145.35, 138.32, and 23.12 ppm assigned to carbon atoms of N-C=C-N and methyl group for the isomer 4-acetyl-2-(4-chlorophenyl)-1,2-dihydro-benzo[*g*]quinoxaline. The carbon signals due to the naphthalene and 4-chlorophenyl rings appeared in the region at δ 138.12–127.56 ppm, while the carbon signal of carbonyl (C=O) group was observed at δ 174.56 ppm. Moreover, reaction of 2-(4-chlorophenyl)-3,4-dihydro-benzo[g]quinoxaline (**2**) with α, β-unsaturated ester; namely, 3-(4-hydroxy-3-methoxy)phenyl-2-cyano acrylate in ethanol in the presence of triethyl amine as a base catalyst yielded ethyl 3-[3-(4-chlorophenyl)-1,2-dihydro-benzo[*g*]quinoxaline-1-yl]-2-cyano-3-aryl acrylates (**5**).

Halogenation of 2,3-napthalene diamine (**1**) with bromine in glacial acetic acid yielded the corresponding 1,4-dibromo naphthalene-2,3-diamine (**6**). Treatment of compound **6** with 4-chlorophenacyl bromide in methanol in the presence of fused sodium acetate under reflux was expected to give structure **7**, but instead 1,4-dibromo-2-(4-chlorobenzoylmethyl)amino-3-amino-naphthalene (**8**) was obtained. Finally, cyclization of 1,4-dibromo-2-(4-chloro-benzoylmethyl)amino-3-amino-naphthalene (**8**) with fused piperidine led to the formation of 1-(5,10-dibromo-3-(4-chlorophenyl)benzo[g]4thenone4ne-1(2*H*)-yl)4thenone (9, Scheme 2).

### 2.2. Biology

#### 2.2.1. Cytotoxic Activity against Breast MCF-7 Cancer Cell Line

The effect of the prepared benzo[*g*]quinoxaline derivatives **2**–**9** on the viability of breast MCF-7 cancer cell line was studied using MTT assay. The cytotoxicity was assessed using Dox as positive reference drug. Treatment of the MCF-7 cell line with different concentration of benzo[*g*]quinoxaline derivatives revealed that some of the tested compounds displayed good cytotoxic activity against MCF-7 cells as concluded from their IC_50_ values presented in Table 1. Benzo[*g*]quinoxaline molecule **3** exhibited the highest cytotoxic activity within the tested molecules against MCF-7 cells with IC_50_ value of 2.89 µM compared with Dox (IC_50_ = 2.01 µM). Moreover, benzo[*g*]quinoxaline derivative **9** with dibromo substitution (IC_50_ = 8.84 µM) showed cytotoxic activity more than benzo[*g*]quinoxaline **4** (IC_50_ = 16.22 µM), which does not have dibromo substitution; this might indicate that bromo-substitution plays an important role in anti-cancer activity of benzo[*g*]quinoxalines.

#### 2.2.2. In Vitro Topoisomerase IIβ Assay

**Figure 3:**
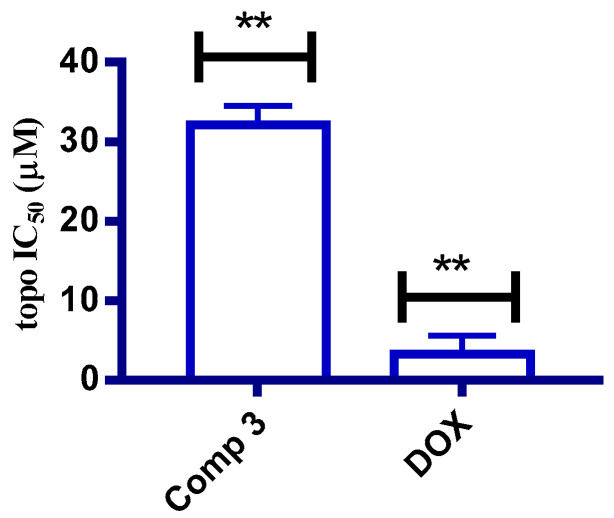
Graphical representation for comparison of topo IIβ IC_50_ of compound **3** and Dox; topo II was performed using four doses protocol for compound **3** and Dox, and the concentration that induced 50% maximum inhibition of topo II was determined from sigmoidal dose–response. Data are expressed as the mean ± SD (*n* = 3 experiments) and statistical comparisons were carried out using one-way analysis of variance (ANOVA) followed by Tukey’s multiple comparison test (** *p* < 0.01). **-Significant.

DNA synthesis has been regarded as one of the most effective targets for the development of novel anticancer agents with apoptosis induction [28]. In addition, several reported experimental results showed that compounds having polycyclic molecular skeleton appear to give the optimal base pair intercalation and enzyme binding with human DNA, leading to reading errors during the replication step [19]. To obtain a further insight into the mechanism of cell proliferation inhibition activity, benzo[*g*]quinoxaline molecule **3** was investigated for topoisomerase IIβ (topo IIβ) inhibitory activity using the Human DNA topo IIβ ELISA Kit # MBS942146. The results revealed that the tested benzo[*g*]quinoxaline molecule **3** had a good inhibitory activity against topo IIβ at submicromolar level with IC_50_ = 32.16 µM compared with value of 3.31 µM for Dox, a known potent topo IIβ inhibitor (Figure 3).

#### 2.2.3. Cell Cycle Analysis

**Figure 4:**
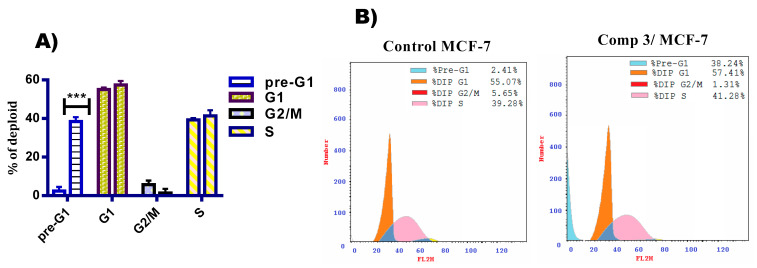
(**A**) Graphical representation of percentages of cells in pre-G1, G1, G2/M, and S cell cycle phase in MCF-7 cells treated with compound **3** (2.89 µM) for 48 h. (**B**) Effect of compound **3** (2.89 µM) on cell cycle distribution in MCF-7 cells for 48 h and analyzed by flow cytometry after staining with DNA fluorochrome propidium iodide; most of the compound **3** treated cells were in pre-G1 phase (apoptosis). Data are expressed as the mean ± SD (*n* = 3 experiments) and statistical comparisons were carried out using one-way analysis of variance (ANOVA) followed by Tukey’s multiple comparison test (*** *p* < 0.001). ***-Highly significant.

Cell cycle analysis was conducted to determine the mechanism of cytotoxic activity of the most active molecule against MCF-7 cells by using DNA flow cytometric analysis [29]. Compound **3** was tested at its IC_50_ concentration dose value. The results showed that the tested compound **3** increased the percentage of cell population at G1 and S phases compared to the untreated control. Moreover, the results showed that compound **3** increased the percentage of cells at the pre-G1 phase from 2.41% to 38.24%, compared to the untreated control (Figure 4).

#### 2.2.4. Annexin V/FITC Apoptosis Staining Assay

**Figure 5:**
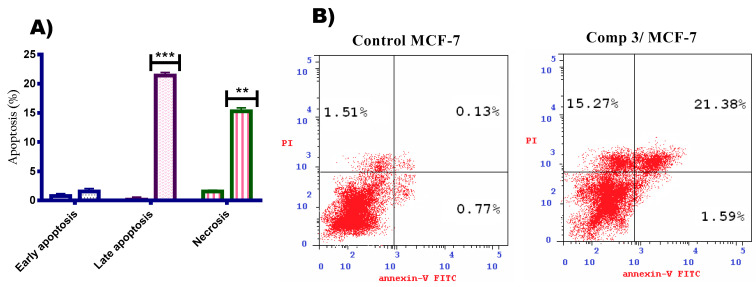
(**A**) Graphical representation of the effect of compound **3** on induction of apoptosis in MCF-7 cell line. (**B**) Effect of compound **3** on induction of apoptosis in MCF-7 cell line. Data are expressed as the mean ± SD (*n* = 3 experiments) and statistical comparisons were carried out using one-way analysis of variance (ANOVA) followed by Tukey’s multiple comparison test (** *p* < 0.01, and *** *p* < 0.001). The lower left quadrant shows live cells; the lower right quadrant shows early apoptotic cells; the upper right quadrant shows late apoptotic cells; and the upper left quadrant shows necrotic cells.

Apoptosis staining assay was performed to detect the effect of compound **3** on the percentage of early and late apoptosis in the breast MCF-7 cancer cell line [30]. As shown in Figure 5, compound **3** increases the apoptosis percentage at the early stage from 0.77% to 1.59% compared with the untreated control. Additionally, the apoptosis percentage at late stage was increased from 0.13% to 21.38% compared with the untreated control. Therefore, compound **3** can be considered an apoptosis inducer in the breast MCF-7 cell line.

#### 2.2.5. Bax and Bcl2 Enzyme Assay

**Figure 6:**
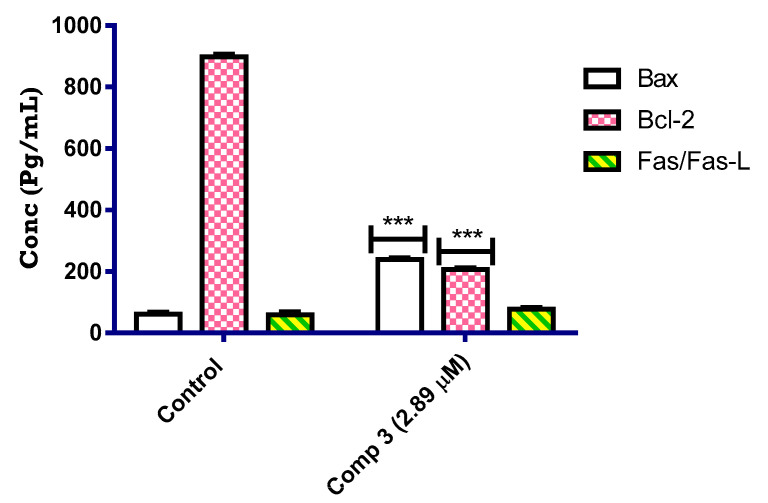
Effect of compound **3** on apoptosis related proteins, Bax, Bcl2, and Fas/Fas-L in MCF-7 cells. The graph represents the increased expression of Bax and decreased expression of Bcl2, during apoptotic event that occurred after the treatment of compound **3** (2.89 µM) in MCF-7 cells and was determined by ELISA assay. Data was expressed as mean ± SD (*n =* 3 experiments) and statistical comparisons were carried out using one-way analysis of variance (ANOVA) followed by Tukey’s multiple comparison test (*** *p* < 0.001).

In order to investigate the molecular pathway involved in apoptosis, compound **3** was screened for its ability to activate pro-apoptotic protein; Bax and downregulate anti-apoptotic protein; Bcl2 [31]. The MCF-7 cancer cell line was treated with compound **3** at its IC_50_ value (µM) and the level of expression of proteins involved in apoptosis was quantized using ELISA assay. From the results presented in Figure 6, compound **3** increased the expression of Bax in MCF-7 cancer cells by 3.89-fold compared with the untreated control. In addition, compound **3** resulted in downregulation of the anti-apoptotic protein Bcl2 by 4.35-fold compared with the untreated control. Therefore, it can be concluded that compound **3** can induce the intrinsic pathway of apoptosis by activation of Bax and downregulation of Bcl2.

#### 2.2.6. Molecular Docking Study

**Figure 7:**
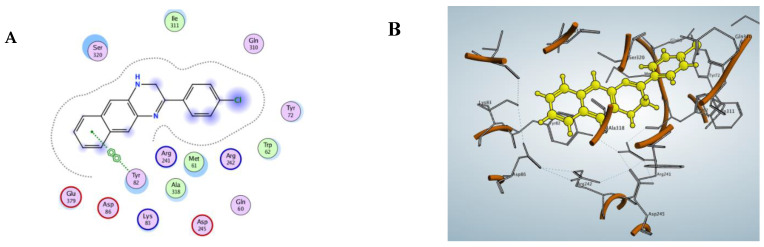
(**A**) 2D binding mode of compound **3** docking in the active site of topoisomerase II (PDB entry: 1ZXM), illustrating one π–π interaction with Tyr 82, hydrogen atoms were omitted for clarity. (**B**) 3D binding mode of compound **3** (rendered in balls and stick mode and colored by yellow) in the active site of topoisomerase II (PDB entry: 1ZXM), hydrogen atoms were omitted for clarity.

Molecular docking study was performed to investigate its plausible binding interaction with the key amino acid in the active site of topoisomerase II. Benzo[*g*]quinoxaline molecule **3** was docked with the crystal structure of topoisomerase II (PDB code 1ZXM). As can be seen, the molecular interaction of compound **3** in Figure 7 revealed π–π interaction with the target protein. In addition, the hydrophobicity induced by compound **3** resulted in docking score of −8.28 kcal/mol.

## 3. Experimental

### 3.1. Chemistry

The melting point of the synthesized derivatives was determined with electro thermal melting point apparatus and has not been corrected. The IR data were obtained on a Jasco FTLIR 6100 infrared spectrometer using KBr disc technique. ^1^H-NMR (400 MHz) and ^13^C-NMR (100 MHz) spectra were run with a Bruker 400 DRX-Avance NMR spectrometer, and measured using tetramethylsilane (TMS) as the internal standard. The mass spectra were measured with a Finnigan MATSSQ-7000 mass spectrometer. Elemental micro-analyses were carried out at the micro analytical unit, Central Services laboratory, National Research Centre, Dokki, Cairo, Egypt, using Vario Elemental and were found to be within ± 0.5% of the theoretical values. All reagents were obtained from Aldrich Chemical Company and used as supplied.

#### 3.1.1. General Procedure for the Synthesis of Compounds **2** and **8**

A mixture of naphthalene-2,3-diamine or 1,4-dibromo-naphthalene-2,3-diamine (0.01 mol), 4-chlorophenacyl bromide (0.01 mol) and fused sodium acetate (0.02 mol) in 30 mL methanol was heated under reflux for 2 h. The reaction mixture was cooled, poured into water with stirring, and the formed precipitate was filtered, washed with water, dried, and recrystallized from ethanol to give **2** and **8**.

##### 3-(4-Chlorophenyl)-1,2-dihydrobenzo[*g*]quinoxaline (**2**)

Yellow crystals, yield 73%, m.p. 195–197 °C. IR (KBr) ν_max_ = 3225 (NH), 1632 (C=N), 1603, 1582 (C=C) cm^−1^. ^1^H-NMR (DMSO-*d_6_*): δ 4.16 (d, 2H, NCH_2_), 7.34 (s, 1H, olefinic CH), 7.51–8.38 (m, 9H, Ar-H and NH), 8.73 (s, 2H, H-5 and H-10 of benzo quinoxaline), 9.58 (s, 1H, NH-C=) ppm. ^13^C-NMR (DMSO-d_6_): δ 151.02, 150.61, 150.50, 148.01, 145.33, 139.37, 138.33, 138.14, 136.19, 135.37, 134.21, 133.77, 130.18, 129.86, 129.48, 128.90, 128.80, 128.70, 128.01, 127.73, 127.65, 127.53, 127.04, 37.72 ppm carbons of benzo quinoxaline. MS (*m*/*z* %) = 294 (M^+^+2), 292 (M^+^, 27.22), 291 (14.11), 290 (50.00), 281 (4.81), 265 (2.79), 255 (8.50), 234 (3.39), 229 (2.93), 211 (2.78), 183 (2.81), 159 (3.30), 153 (2.94), 152 (3.90), 146 (1.99), 145 (3.70), 139 (2.84), 137 (4.77), 132 (3.20), 128 (5.12), 127 (12.02), 126 (100), 125 (7.35), 123 (5.43), 114 (3.7), 113 (2.58), 111 (4.21), 110 (2.63), 103 (6.63), 102 (3.96), 100 (3.10), 99 (3.73), 91 (2.03), 87 (8.25), 85 (2.91), 83 (2.75), 77 (8.84), 76 (1.57), 75 (17.28), 74 (5.40), 71 (2.15). 70 (4.83), 63 (10.15), 62 (2.60), 60 (3.59), 57 (5.86), 55 (2.23), and 54 (4.99). Anal. Calcd. for C_18_H_13_N_2_Cl (292.76): C, 73.85; H, 4.48; N, 9.57. Found: C, 73.58; H, 4.21; and N, 9.33.

##### 2-(3-Amino-1,4-dibromonaphthalen-2-ylamino)-1-(4-chlorophenyl)ethanone (**8**)

Orange crystals, yield 63%, m.p. 225–227 °C. IR (KBr) ν_max_ = 3410, 3235, 3151 (NH_2_, NH), 1716 (C=O), 1603, 1587, 1583 (C=C) cm^−1^. ^1^H-NMR (DMSO-*d_6_*): δ 4.79 (s, 1H, NCH_2_), 4.95 (s, 1H, NCH_2_), 5.75–5.81 (br. s, 3H, NH_2_ and NH), 7.55–8.45 (m, 8H, Ar-H) ppm. ^13^C-NMR (DMSO-*d_6_*): δ 195.66 (C=O), 140.63, 133.21, 132.75, 132.39, 131.79, 131.60, 131.52, 130.45, 129.68, 129.38, 90.1, 25.69 ppm. MS (*m*/*z*, %) = 466 (M^+^, unstable), 422 (1.66), 421 (2.37), 419 (2.19), 417 (4.69), 416 (2.03), 408 (2.17), 343 (1.65), 341 (2.28), 340 (0.74), 337 (0.99), 289 (1.09), 288 (0.77), 287 (0.52), 283 (1.24), 260 (0.75), 259 (0.66), 227 (1.54), 218 (2.45), 217 (0.67), 199 (1.02), 158 (1.61), 156 (0.72), 141 (27.31), 140 (3.87), 139 (100), 127 (1.01), 125 (8.71), 124 (3.61), 113 (15.92), 112 (2.83), 111 (58.46), 110 (3.44), 103 (2.62), 100 (1.23), 99 (2.17), 95 (1.69), 89 (6.97), 88 (3.30), 86 (1.47), 85 (3.62), 82 (48.28), 81 (28.10), 80 (50.72), 79 (23.57), 77 (2.20), 76 (7.40), 75 (38.29), 74 (13.23), 65 (1.44), 64 (4.64), 63 (8.65), 62 (7.58), 51 (4.27), and 50 (13.73). Anal. Calcd. for C_18_H_13_N_2_Br_2_ClO (468.75): C, 46.14; H, 2.80; and N, 5.98. Found: C, 46.01; H, 2.47; and N, 5.79.

#### 3.1.2. General Procedure for the Synthesis of 2-(4-Chlorophenyl)benzo[g]quinoxaline (**3**)

A solution of compound **2** in acetic anhydride (25 mL) was refluxed for 2 h, then cooled and poured into ice-cold water with stirring. The reaction mixture was left for 24 h and the formed solid was filtered off, washed with water, dried, and crystallized from a suitable solvent to give compound **3**.

Colorless crystals, yield 66%, m.p. 168–170 °C. IR (KBr) ν_max_ = 1656 (C=N), 1605, 1583 (C=C) cm^−1^. ^1^H-NMR (DMSO-*d*_6_): δ 7.68–8.46 (m, 8H, Ar-H), 8.81 (s, 2H, H-5 and H-10 of quinoxaline ring), 9.66 (s, 1H, CH=N) ppm. ^13^C-NMR (DMSO-*d*_6_): δ 150.49, 145.34 (C=N), 138.33, 138.14, 136.19, 135.38, 134.21, 133.78, 129.86, 129.71, 128.90, 128.80, 128.01, 127.73, 127.63, 127.53 (C-aromatic of benzoquinoxaline ring) ppm. MS (*m*/*z*, %) = 292 (M^+^+2, 32.50), and 290 (M^+^, 100). Anal. Calcd. for C_18_H_11_N_2_Cl (290.75): C, 74.36; H, 3.81; and N, 9.63. Found: C, 74.11; H, 3.53; and N, 9.49.

#### 3.1.3. General Procedure for the Synthesis of 1-(3-(4-Chlorophenyl)benzo[g]quinoxalin-1(4H)-yl)ethanone (**4**)

A suspension of compound **2** (0.01 mol) in acetonitrile (30 mL), acetic anhydride (0.02 mol) was added. The reaction mixture was heated under reflux for 12 h; after, the reaction mixture was cooled to room temperature and the formed precipitate was filtered off, dried, and then crystallized from absolute ethanol to give compound **4**.

Pale yellow crystals, yield 61%, m.p. 171–173 °C. IR (KBr) ν_max_ = 3226 (NH), 1698 (C=O), 1632 (C=N), 1605, 1583 (C=C) cm^−1^. ^1^H-NMR (DMSO-*d_6_*): δ 1.12 (s, 3H, COCH_3_), 2.18 (s, 3H, COCH_3_), 3.92 (s, 2H, NCH_2_), 7.35–7.38 (m, 5H, Ar-H and H-olefinic), 7.94–8.12 (m, 4H, Ar-H), 8.48 (s, 2H, H-5 and H-10 of quinoxaline ring), 9.32 (s., 1H, NH) ppm. ^13^C-NMR (DMSO-*d*_6_): δ 174.56 (C=O), 150.52, 145.35, 138.32, 138.12, 136.19, 135.36, 134.21, 133.77, 129.82, 129.72, 128.90, 128.81, 128.01, 127.72, 127.68, 127.56 (C- aromatic and benzo quinoxaline ring), 44.48 (NCH_2_), 23.79, 23.12 (CH_3_ of two isomers) ppm. MS (*m*/*z*, %) = 334 (M^+^, unstable), 333 (0.12), 292 (12.64), 291 (9.07), 290 (34.49), 289 (3.86), 263 (1.78), 256 (1.14), 255 (5.64), 228 (1.23), 227 (1.31), 175 (1.49), 168 (1.00), 153 (2.20), 152 (1.52), 151 (2.29), 145 (2.82), 139 (3.60), 137 (2.16), 132 (2.50), 131 (1.13), 127 (10.07), 126 (95.02), 114 (4.55), 113 (2.84), 112 (6.69), 111 (1.56), 102 (1.68), 101 (1.96), 100 (2.53), 98 (1.71), 97 (2.80), 87 (2.32), 86 (18.28), 85 (53.41), 84 (100), 77 (1.37), 76 (5.23), 75 (6.05), 74 (3.08), 70 (10.37), 69 (5.25), 60 (13.44), 57 (16.07), 56 (27.52), and 55 (8.13). Anal. Calcd. for C_20_H_15_N_2_ClO (334.80): C, 71.75; H, 4.52; and N, 8.37. Found: C, 71.57; H, 4.23; and N, 8.12.

#### 3.1.4. General Procedure for the Synthesis of Ethyl 3-(3-(4-Chlorophenyl)benzo[g]quinoxalin-1(4H)-yl)-2-cyano-3-(4-hydroxy-3-methoxyphenyl)acrylate (**5**)

A mixture of **2** (0.01 mol), ethyl 3-(4-hydroxy-3-methoxy)phenyl-2-cyano acrylates (0.01 mol) in absolute ethanol (30 mL) in presence of triethyl amine (1 mL) was refluxed for 3 h. The reaction mixture was cooled, poured into water, and neutralized with dilute hydrochloric acid (2%). The formed precipitate was separated by filtration and crystallized from ethanol to give **5**.

Pale yellow crystals, yield 71%, m.p. 146–148 °C. IR (KBr) ν_max_ = 3432 (br. OH), 3262 (NH), 2221 (CN), 1727 (C=O), 1605, 1583 (C=C), 1121, 1096, 1032 (C-O) cm^−1^. ^1^H-NMR (DMSO-*d_6_*): δ 1.23 (t, 3H, CH_3_), 3.76 (s, 3H, OCH_3_), 4.23 (q, 2H, OCH_3_), 6.88 (d, 2H, Ar-H), 7.56–7.70 (m, 7H, Ar-H), 8.08–8.74 (m, 5H, Ar-H), 9.59 (s, 1H, NH), 10.51 (br. s, 1H, OH) ppm. ^13^C-NMR (DMSO-*d_6_*): δ 163.11 (C=O), 155.45, 153.13 (C-O), 150.51, 148.26, 145.35 (C-N), 138.33, 138.14, 136.19, 135.37, 134.21, 133.77, 129.86, 129.71, 128.90, 128.81, 128.01, 127.73, 127.66, 127.60, 127.54, 123.30, 117.13, 116.48, 114.55 (CN), 62.45 (OCH_2_), 56.03 (COCH_3_), 14.53 (CH_3_) ppm. MS (*m*/*z*, %) = 537.50 (M^+^, unstable), 324 (5.43), 323 (4.86), 322 (20.74), 320 (6.06), 308 (2.00), 307 (1.95), 306 (5.00), 305 (2.06), 303 (3.03), 302 (1.39), 293 (1.81), 292 (35.91), 291 (19.80), 290 (81.90), 289 (4.58), 281 (1.35), 280 (1.06), 278 (14.84), 268 (2.56), 264 (2.62), 256 (2.09), 255 (4.33), 248 (1.32), 247 (14.94), 233 (2.35), 222 (1.53), 202 (1.29), 191 (1.38), 181 (1.40), 179 (1.10), 175 (1.17), 170 (2.70), 161 (1.06), 152 (3.30), 150 (1.58), 146 (1.07), 145 (8.40), 141 (4.42), 140 (1.15), 139 (10.94), 138 (3.32), 137 (3.32), 136 (2.80), 131 (2.59), 127 (13.92), 126 (100), 125 (5.02), 121 (1.90), 115 (1.86), 114 (12.23), 111 (2.32), 102 (2.54), 101 (3.07), 100 (5.80), 99 (2.66), 95 (3.10), 77 (3.20), 76 (5.72), 75 (7.99), and 74 (1.90). Anal. Calcd. for C_31_H_24_N_3_ClO_4_ (537.99): C, 69.21; H, 4.50; and N, 7.81. Found: C, 69.02; H, 4.19; and N, 7.67.

#### 3.1.5. General Procedure for the Synthesis of 1-(5,10-Dibromo-3-(4-chlorophenyl)benzo[*g*]quinoxalin-1(2*H*)-yl)ethanone (**9**)

A mixture of compound **8** (0.01 mol) with piperidine (2 mL) was fused on a hot plate for 15–20 min. Then, acetic acid (15 mL) was added with continuous heating under reflux for further 1 h, the reaction mixture left to cool, and then poured into ice-water with stirring. The formed precipitate was filtered, washed with water, dried, and crystalized from ethanol to give compound **9.**

Orange crystals, yield 56%, m.p. 205–207 °C. IR (KBr) ν_max_ = 3267 (NH), 1698 (C=O), 1631 (C=N), 1606, 1588 (C=C) cm^−1^. ^1^H-NMR (DMSO-*d_6_*): δ 1.75 (s, 3H, COCH_3_), 1.94 (s, 3H, COCH_3_), 2.23 (s, 2H, NCH_2_) 6.94–7.99 (m, 8H, Ar-H), 8.01 (s, 1H, H-olefinic), 8.07 (s, 1H, NH) ppm. ^13^C-NMR (DMSO-*d_6_*): δ 160.20 (C=O), 159.70, 148.99, 138.72, 138.53, 130.58, 130.43, 129.49, 129.35, 128.98, 128.84, 128.71, 127.92, 127.84, 127.77, 127.16, 127.06, 126.71, 126.50, 121.81, 121.76, 102.63 (Carbons of benzoquinoxaline and phenyl rings), 44.04 (NCH_2_), 22.58, 22.13 (CH_3_ of two isomer) ppm. MS (*m*/*z*, %) = 490.5 (M^+^, 1.91), 489 (M^+^-1, 1.91), 486 (1.99), 463 (1.11), 454 (3.09), 448 (1.69), 445 (1.38), 442 (1.02), 438 (4.27), 435 (3.39), 431 (1.29), 423 (16.56), 421 (100), 419 (22.67), 418 (88.79), 417 (18.45), 416 (30.72), 408 (1.47), 407 (14.20), 406 (18.05), 405 (5.90), 404 (19.95), 393 (1.60), 391 (1.36), 377 (4.15), 376 (2.15), 362 (2.70), 360 (1.16), 352 (1.93), 351 (1.03), 342 (14.62), 341 (14.03), 340 (34.97), 339 (22.62), 338 (12.35), 329 (2.37), 328 (12.70), 327 (6.22), 326 (51.45), 325 (7.91), 324 (26.73), 314 (1.48), 312 (3.57), 300 (3.00), 299 (4.54), 298 (7.98), 297 (7.35), 296 (2.44), 295 (4.88), 289 (1.18), 281 (2.35), 276 (1.42), 270 (3.18), 268 (2.29), 267 (1.41), 263 (1.43), 261 (5.00), 260 (4.27), 259 (6.76), 258 (11.77), 256 (2.12), 249 (1.62), 248 (1.21), 247 (4.21), 246 (5.63), 244 (4.06), 243 (1.87), 242 (1.80), 239 (4.72), 238 (1.72), 236 (3.54), 235 (6.61), 234 (2.03), 233 (10.87), 232 (4.83), 220 (14.39), 219 (23..83), 218 (36.01), 217 (18.22), 216 (32.50), 210 (8.21), 209 (3.68), 197 (1.62), 196 (2.44), 195 (2.32), 194 (4.42), 193 (2.71), 192 (1.87), 191 (3.94), 190 (7.28), 189 (3.96), 185 (1.92), 184 (1.40), 183 (3.43), 180 (2.05), 179 (9.98), 178 (5.69), 176 (3.12), 175 (3.98), 167 (6.03), 166 (16.21), 165 (7.17), 162 (5.64), 156 (10.45), 155 (3.41), 154 (4.96), 152 (9.51), 150 (5.14), 149 (15.10), 148 (5.44), 144 (4.78), 142 (2.70), 141 (3.18), 140 (26.63), 138 (73.28), 135 (5.82), 134 (3.87), 128 (6.50), 126 (4.73), 125 (11.60), 124 (6.05), 123 (7.73), 116 (4.19), 114 (2.93), 113 (3.08), 112 (18.71), 111 (12.66), 110 (15.70), 109 (15.75), 104 (2.37), 103 (5.56), 100 (8.15), 99 (5.73), 97 (7.16), 96 (2.64), 95 (12.11), 86 (6.52), 85 (51.04), 84 (57.15), 83 (53.99), 76 (6.58), 75 (11.88), 73 (8.25), 72 (5.87), 69 (11.09), 68 (5.04), 62 (13.00), 61 (20.54), 57 (23.60), 56 (35.72), and 55 (14.10). Anal. Calcd. for C_20_H_13_N_2_Br_2_ClO (490.59): C, 48.77; H, 2.66; and N, 5.69. Found: C, 48.68; H, 2.42; and N, 5.61.

### 3.2. Biological Studies

#### 3.2.1. Cytotoxic Activity against Breast MCF-7 Cancer Cell Line

Antitumor activity of the newly prepared benzo[*g*]quinoxaline derivatives **2–9** were carried out against breast MCF-7 cancer cell line using MTT assay method. Cells at density of 1 × 10^4^ were seeded in 96-well plate at 37 °C for 48 h under 5% CO_2_. After incubation, the cells were treated with different concentration of the prepared molecules and incubated for 24 h. MTT dye was added at the end of 24 h of drug treatment and incubated for 4 h at 37 °C. 100 µL of dimethyl sulphoxide was added to each well to dissolve the purple formazan formed. The color intensity of the formazan product, which represents the growth condition of the cells, was quantified using an ELISA plate reader at 570 nm. The experimental conditions were carried out with at least three replicates and the experiments were repeated at least three times.

#### 3.2.2. Topoisomerase IIβ Enzyme Assay

Compound **3** and Dox were evaluated against topoisomerase IIβ for their inhibitory concentration (µM) using four doses concentration according to manufacturer’s instructions [32].

#### 3.2.3. Cell Cycle Analysis Compound **3**

MCF-7 cells (2 × 10^5^/well) were harvested and washed twice in PBS. After that, cells were incubated at 37 °C and 5% CO_2_. The medium was replaced with (DMSO 1% *v*/*v*) containing the tested compound, then incubated for 48 h, washed twice in PBS, fixed with 70% ethanol, rinsed again with PBS, and then stained with DNA fluorochrome PI for 15 min at 37 °C. DNA content was analyzed by flow cytometry on a *FACS Calibur* flow cytometer (Becton and Dickinson, Heidelberg, Germany).

#### 3.2.4. Annexin V FITC/PI Apoptosis Detection Staining Assay

Apoptosis in MCF-7 cells was investigated using fluorescent Annexin V-FITC/PI detection kit by flow cytometry assay. Briefly, MCF-7 cells (2 × 10^5^) after incubation for 12 h. Cells were treated with compound **3** at its IC_50_ concentration for 48 h, then the cells were harvested and stained with Annexin V-FITC/PI dye for 15 min in the dark at 37 °C. Flow cytometry analyses were performed using *FACS Calibur* flow cytometer (Becton and Dickinson, Heidelberg, Germany).

#### 3.2.5. In Vitro ELISA Measurement for the Concentration of Bax, Bcl2, and Fas/Fas-l Proteins

The levels of the apoptotic marker Bax, Bcl-2, and Fas/Fas-l were assessed using ELISA kit. The procedure of the used kits was completed according to the manufacturer’s instructions. Briefly, cell lysates were prepared from control and MCF-7 cells (2.5 × 10^5^) treated with IC_50_ concentration of compound **3**. Then, equal amounts of cell lysates were loaded then probed with specific antibodies. The samples were measured at 450 nm in ROBONEK P2000 ELISA reader. All experiments were performed in duplicate.

#### 3.2.6. Molecular Docking Study

Molecular docking study was performed using MOE software program (MOE 2009.10). The topoisomerase II crystal structure (PDB entry: 1ZXM) was obtained from protein data bank (Supplementary Materials).

## 4. Conclusions

A series of 2,4-disubstituted benzo[*g*]quinoxaline molecules were synthesized, and their in vitro cytotoxic activity were evaluated against the breast MCF-7 cancer cell line using MTT colorimetric assay. The tested molecules revealed good activity against MCF-7 cells. The results of DNA topoisomerase IIβ inhibition activity showed that compound **3** exhibited good inhibition activity against topo IIβ at submicromolar level compared with Dox, a known potent topo IIβ inhibitor. Moreover, these compounds were found to be apoptotic inducers via activation of Bax and downregulation of Bcl2. Therefore, the prepared benzo[*g*]quinoxaline molecules can be considered a scaffold for further optimization to find a more potent cytotoxic agent with better apoptotic inducing activity.

## Figures and Tables

**Table 1:** In vitro cytotoxic activity of benzo[*g*]quinoxaline derivatives **2–5b** and **9** on the MCF-7 breast cancer cell line. Data are expressed as the mean ± SD with *n* = 3 experiments.

Compound No.	IC_50_ (μM)
	MCF-7
**2**	17.24 ± 0.69
**3**	2.89 ± 0.11
**4**	16.22 ± 0.58
**5**	32.14 ± 1.11
**9**	8.84 ± 0.36
Dox	2.01 ± 0.01

## Data Availability

Date is contained in the article.

## Supplementary Materials

The following are available online at https://www.mdpi.com/article/10.3390/ph14090853/s1.
