# Supplementary material for: Evaluation of Synthetic 2,4-Disubstituted-benzo[g]quinoxaline Derivatives as Potential Anticancer Agents
Source: Pharmaceuticals (Basel). 2021 Aug 26;14(9):853. doi: 10.3390/ph14090853 (PMC8466781; doi:10.3390/ph14090853)
Supplement: Supplementary file 1 [file pharmaceuticals-14-00853-s001.zip › pharmaceuticals-1330398-supplementary.pdf]

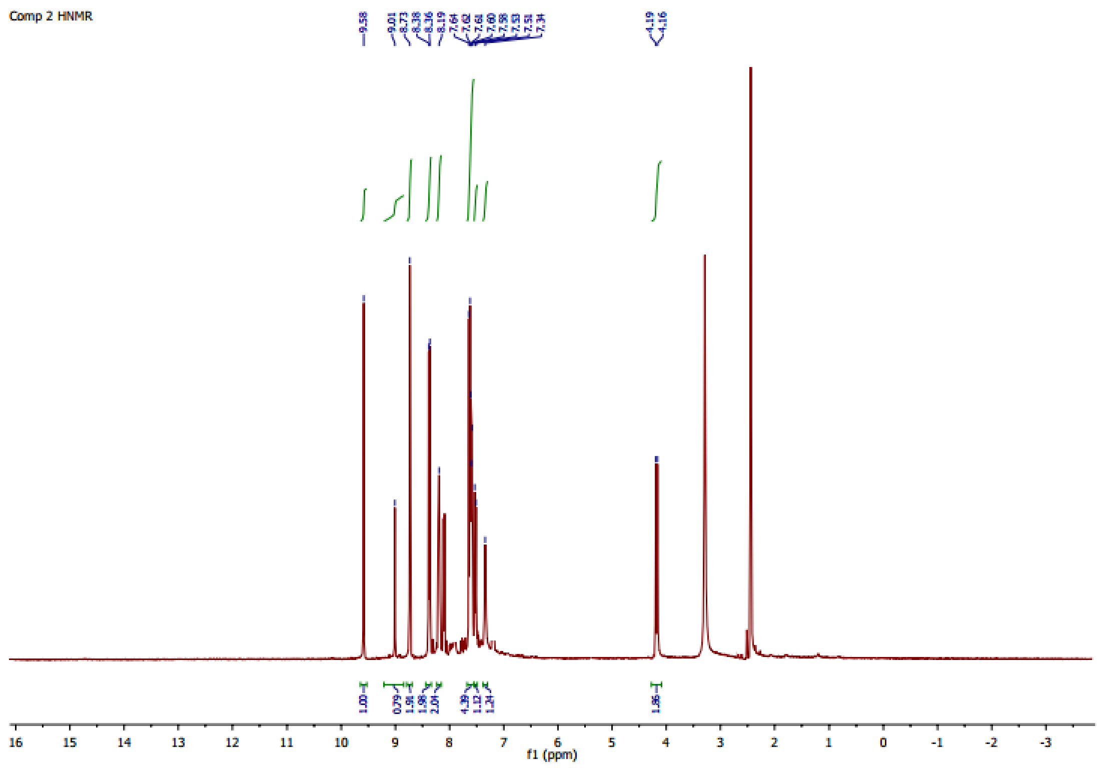

Figure S1. ^{1}H-NMR Spectrum of compound **2.**

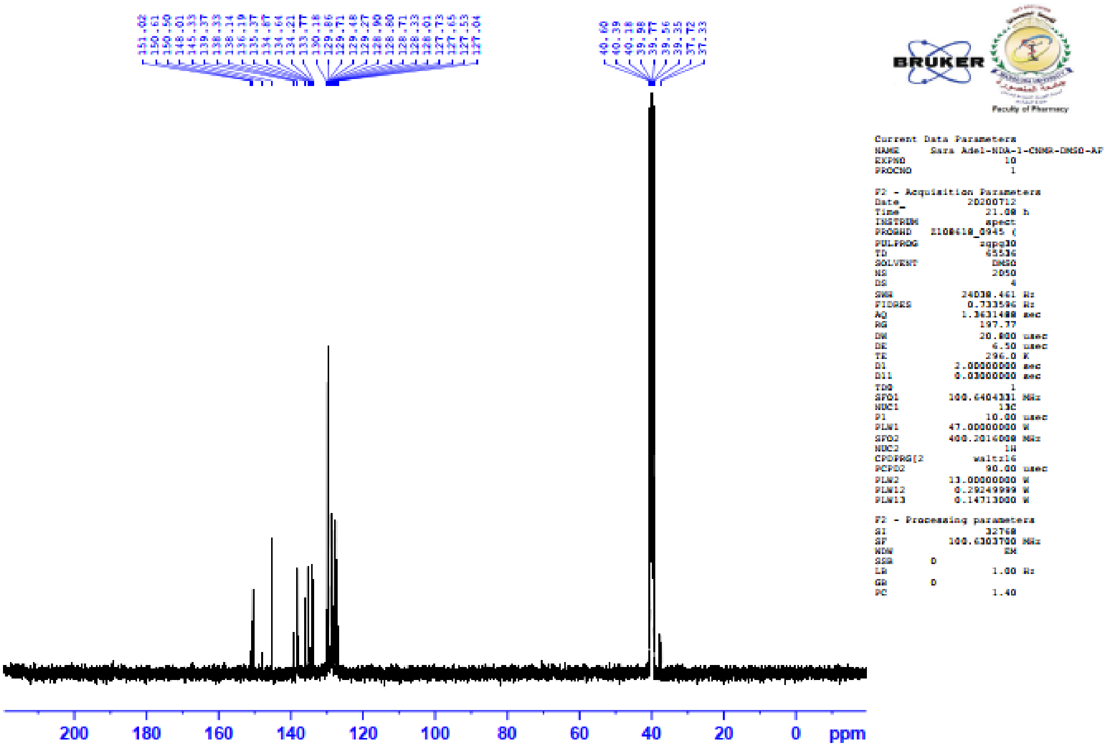

Figure S2. ^{13}C-NMR Spectrum of compound **2**

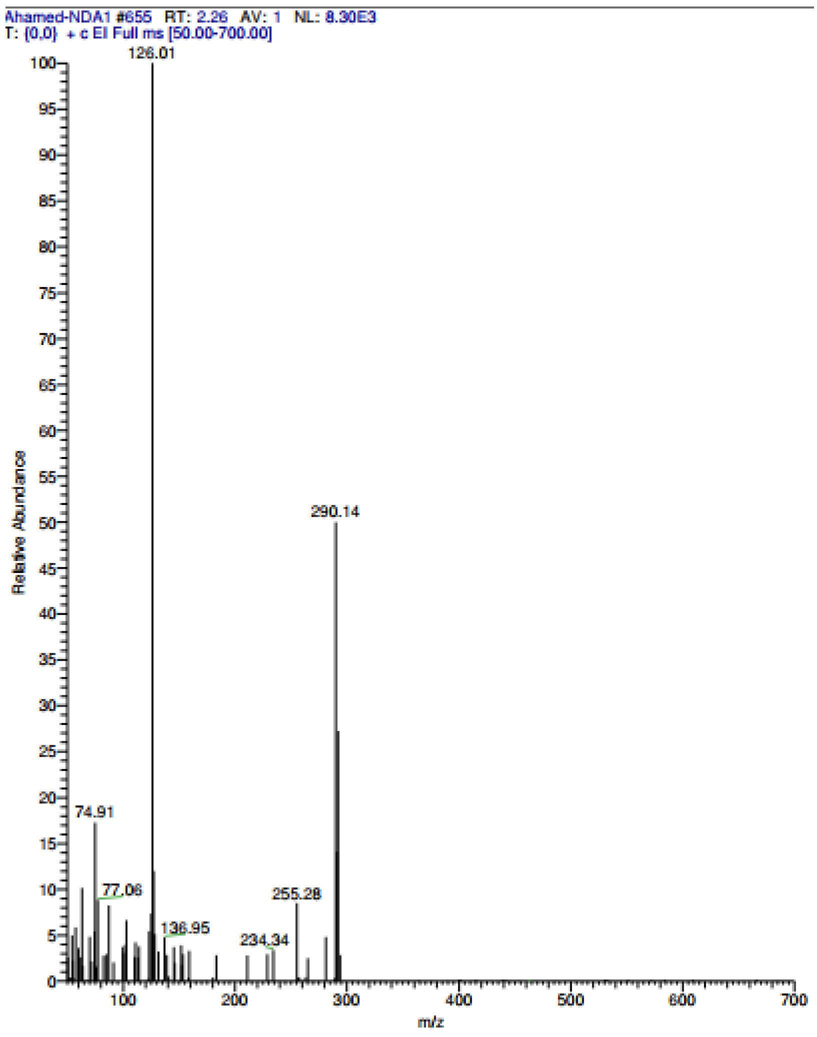

Figure S3. Mass Spectrum of compound **2**

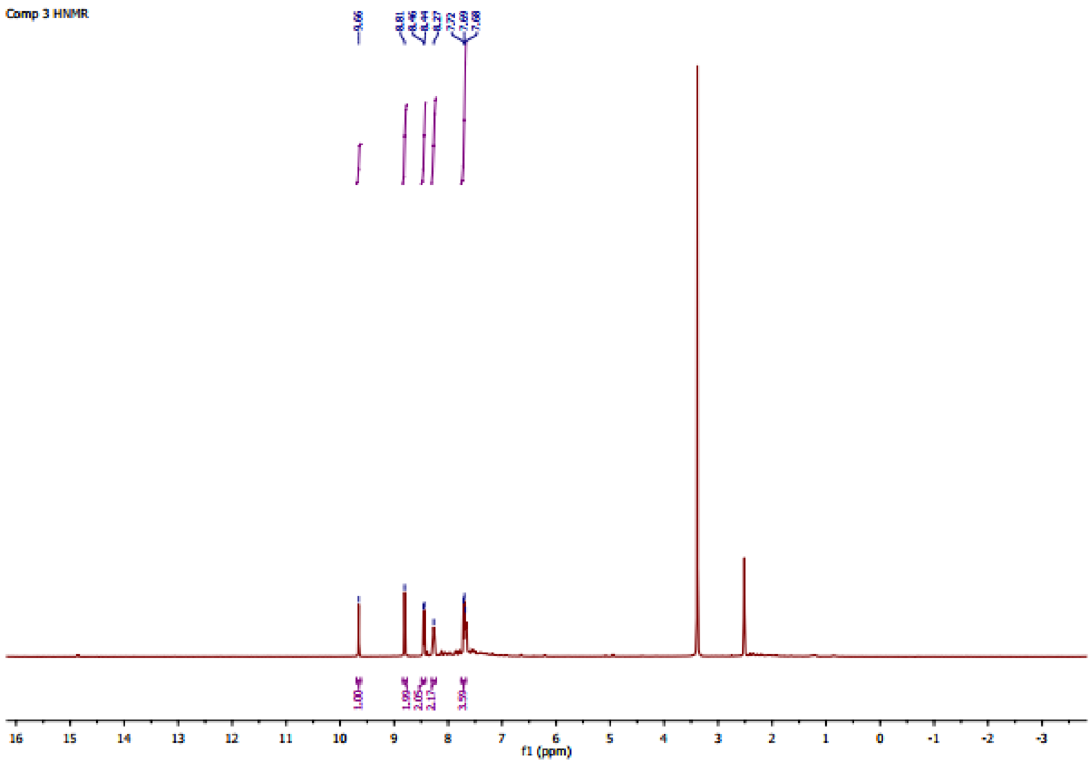

Figure S4. ^{1}H-NMR Spectrum of compound **3**

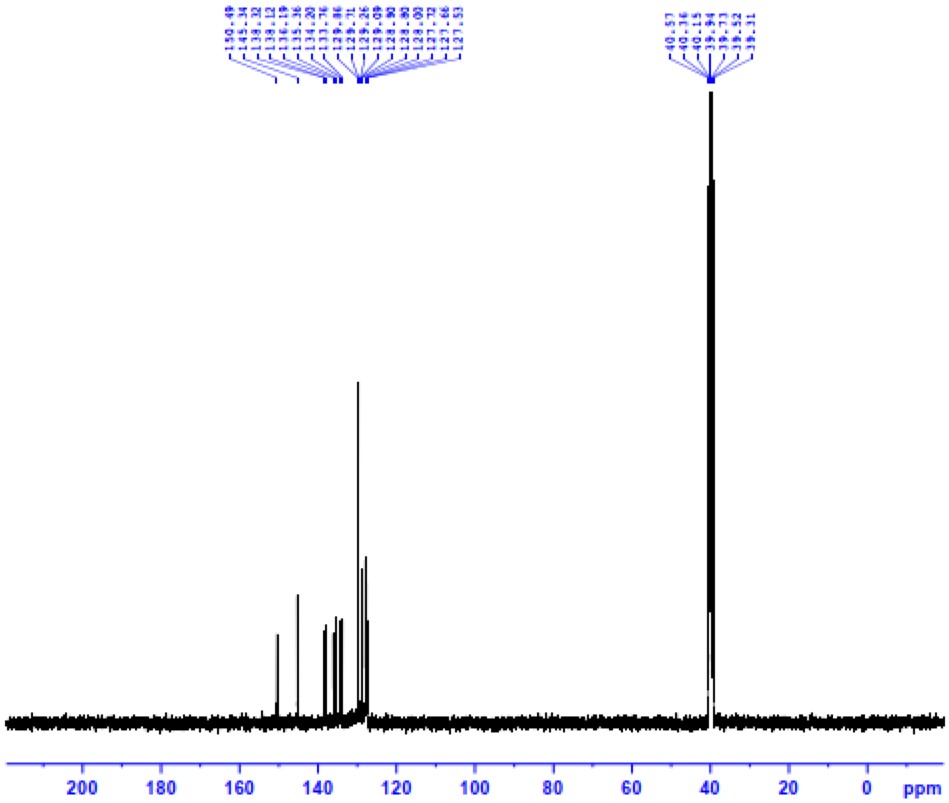

Figure S5. ^{13}C-NMR Spectrum of compound **3**

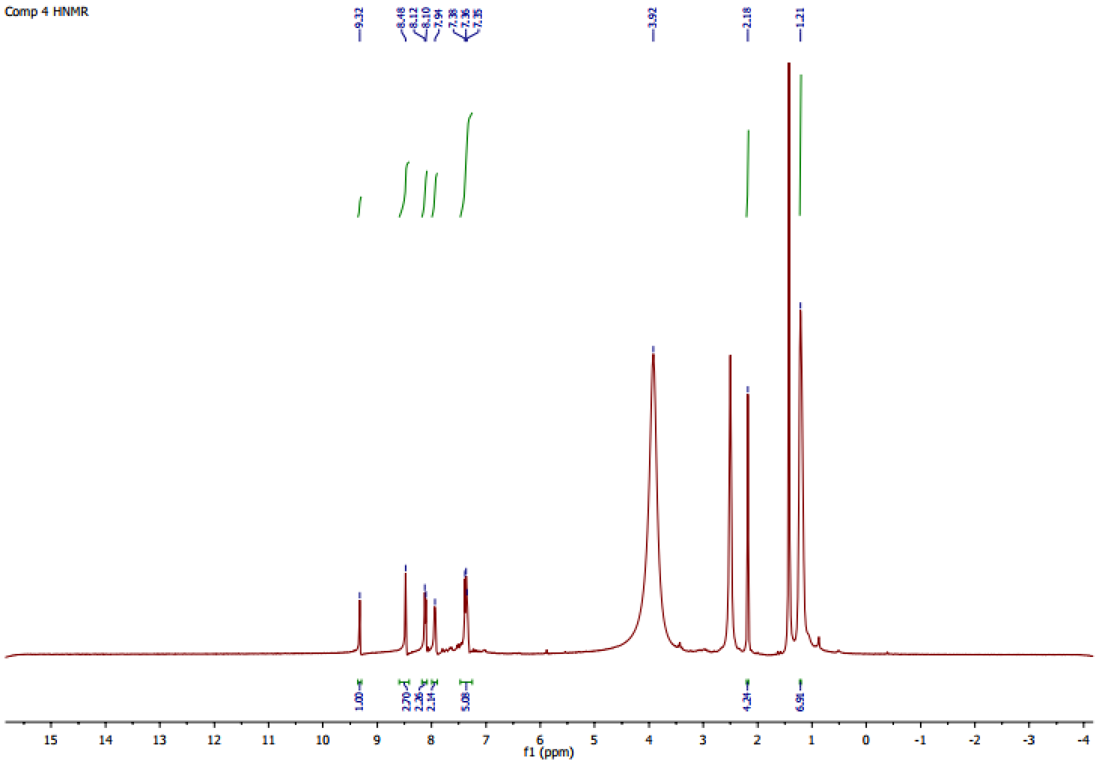

Figure S6. ^{1}H-NMR Spectrum of compound **4**

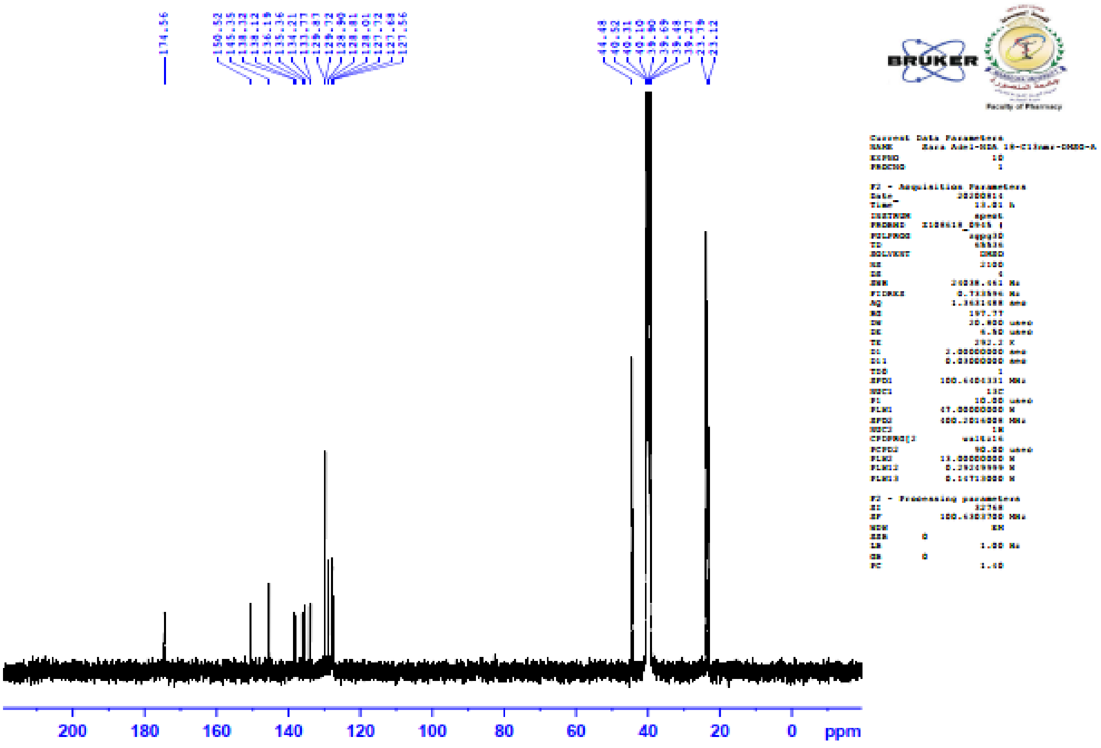

Figure S7. ^{13}C-NMR Spectrum of compound **4**

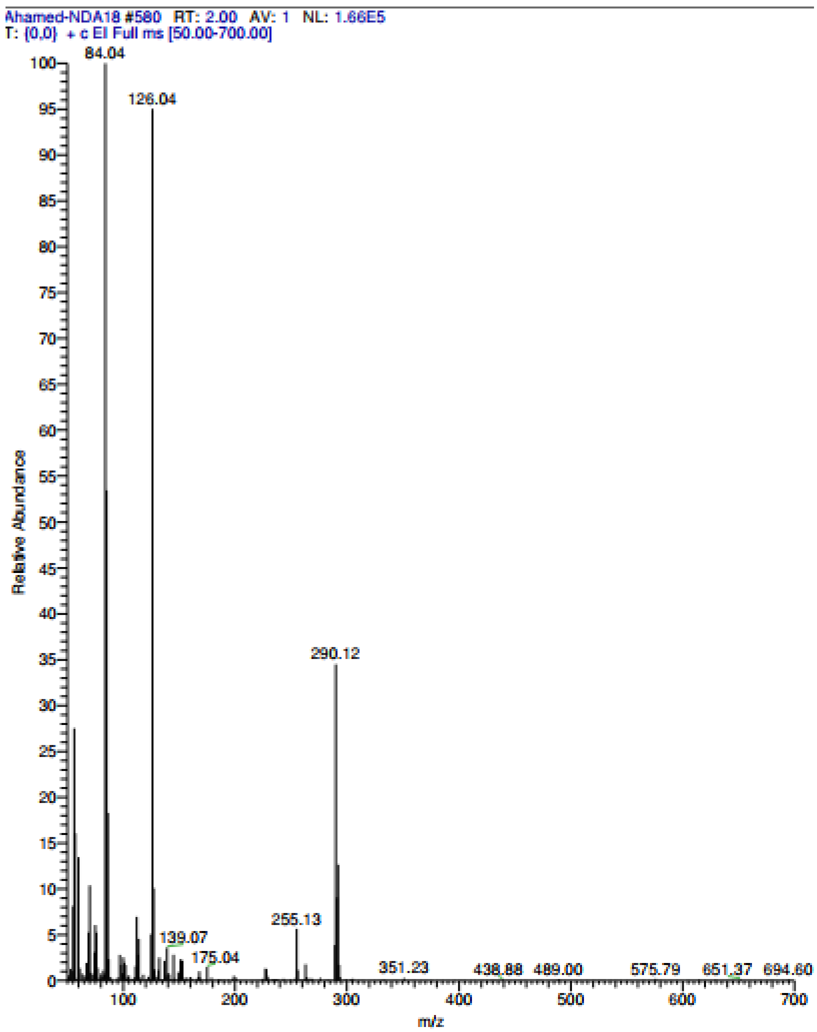

Figure S8. Mass Spectrum of compound **4**

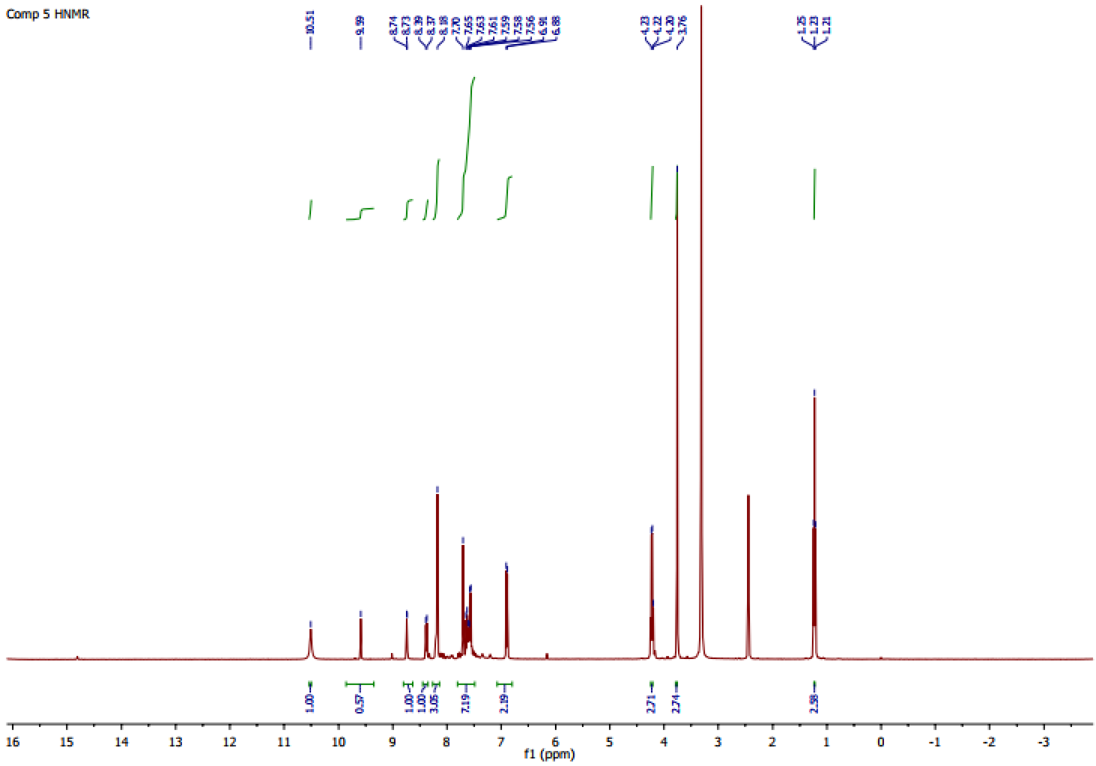

Figure S9. ^{1}H-NMR Spectrum of compound **5**

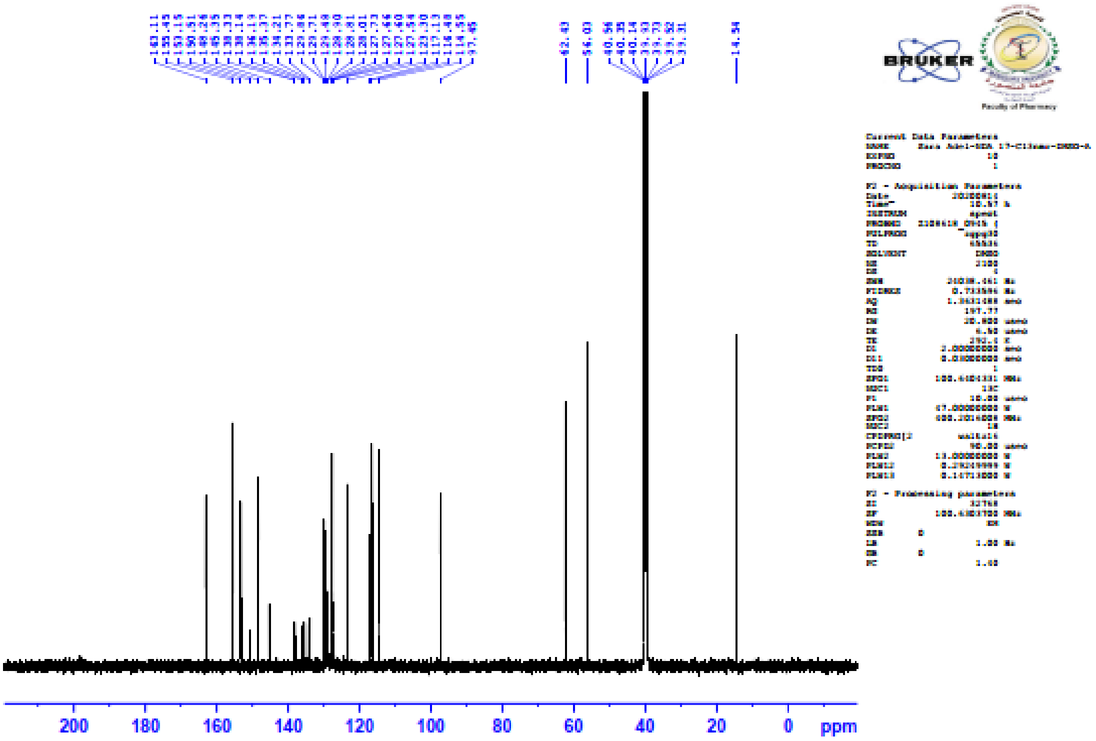

Figure S10. ^{13}C-NMR Spectrum of compound **5**

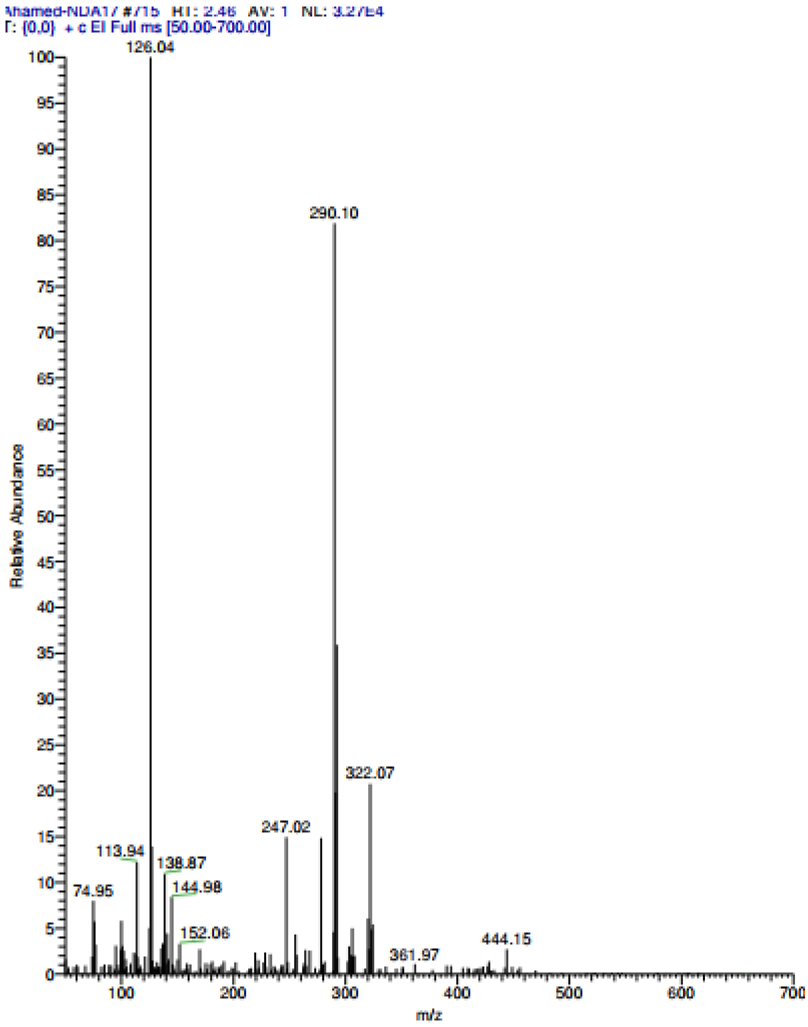

Figure S11. Mass Spectrum of compound **5**

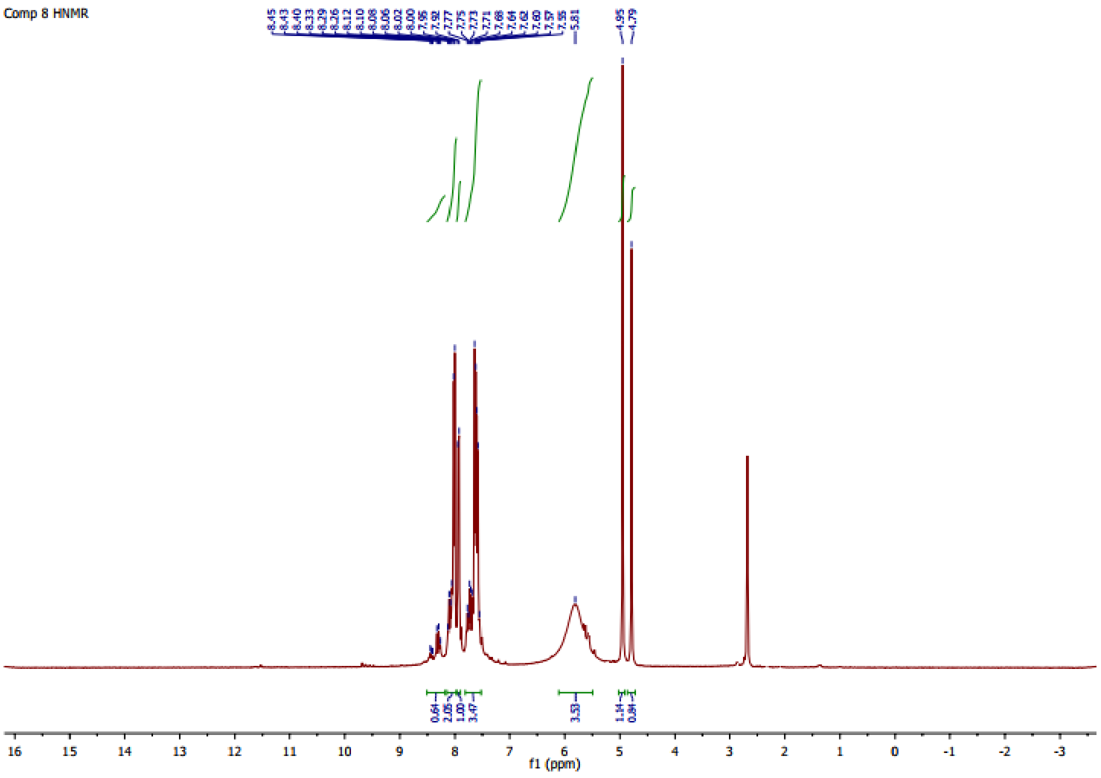

Figure S12. ^{1}H-NMR Spectrum of compound **8**

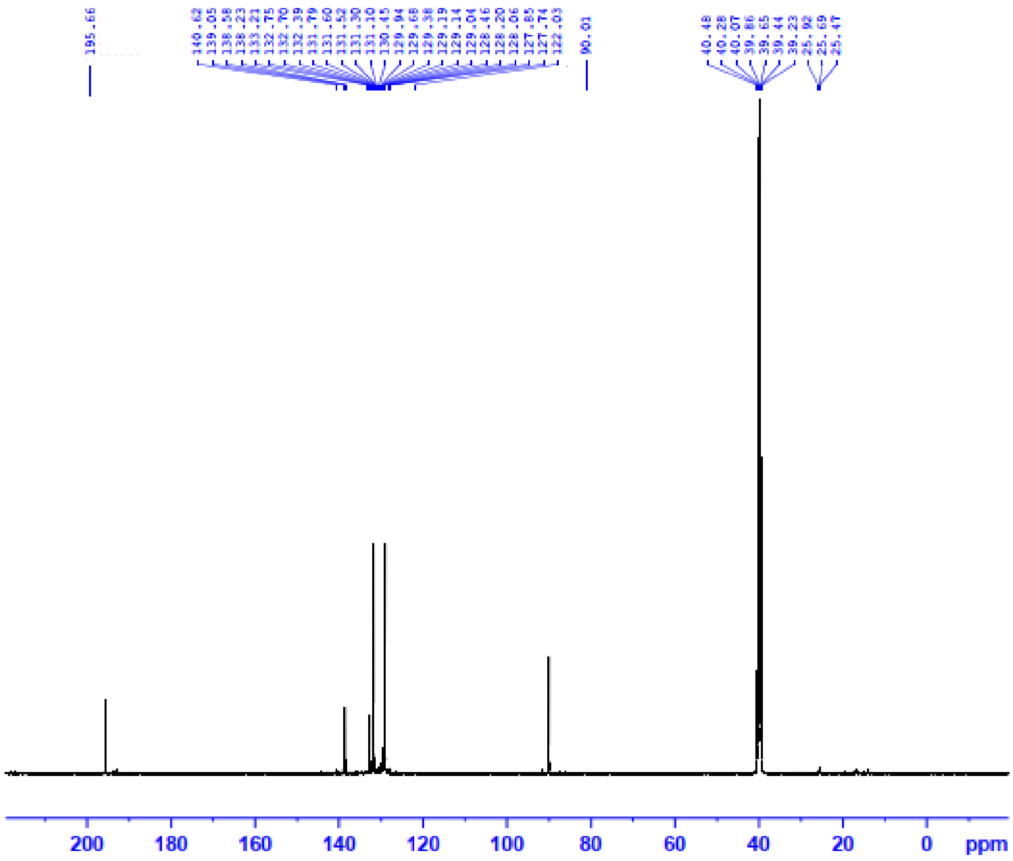

Figure S13. ^{13}C-NMR Spectrum of compound **8**

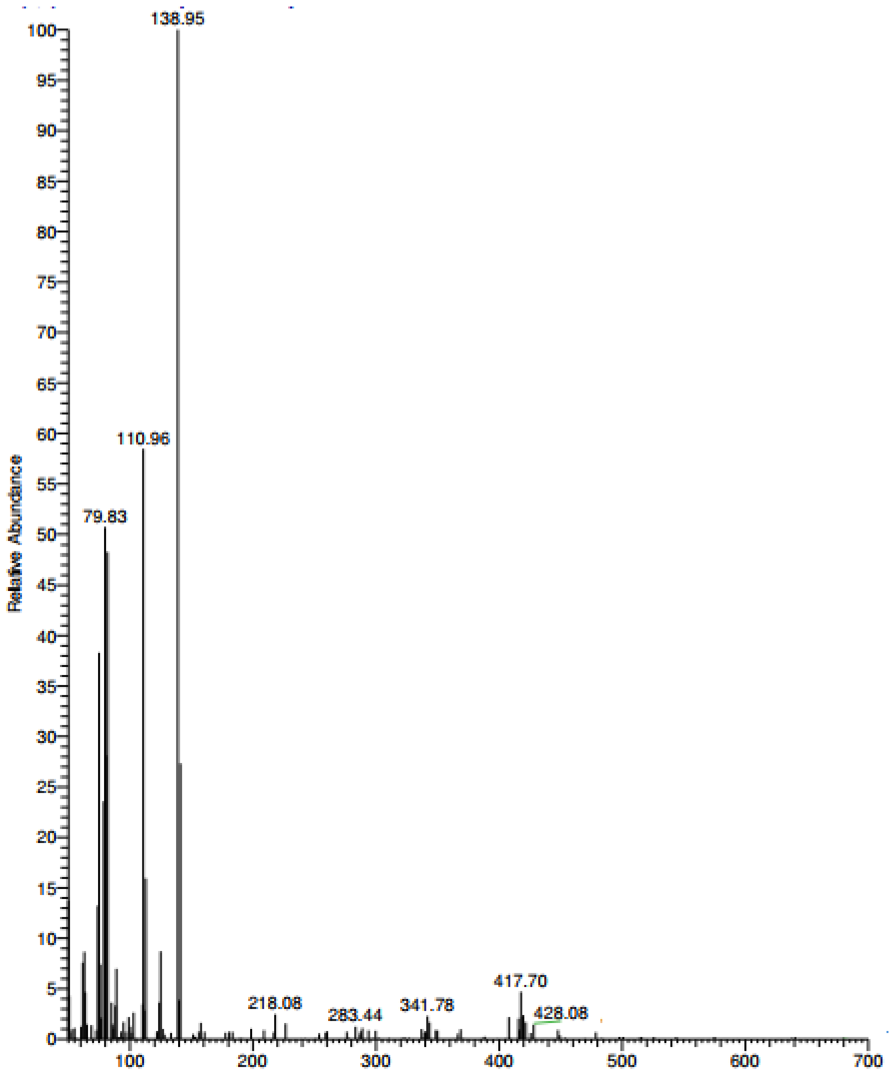

Figure S14. Mass Spectrum of compound **8**

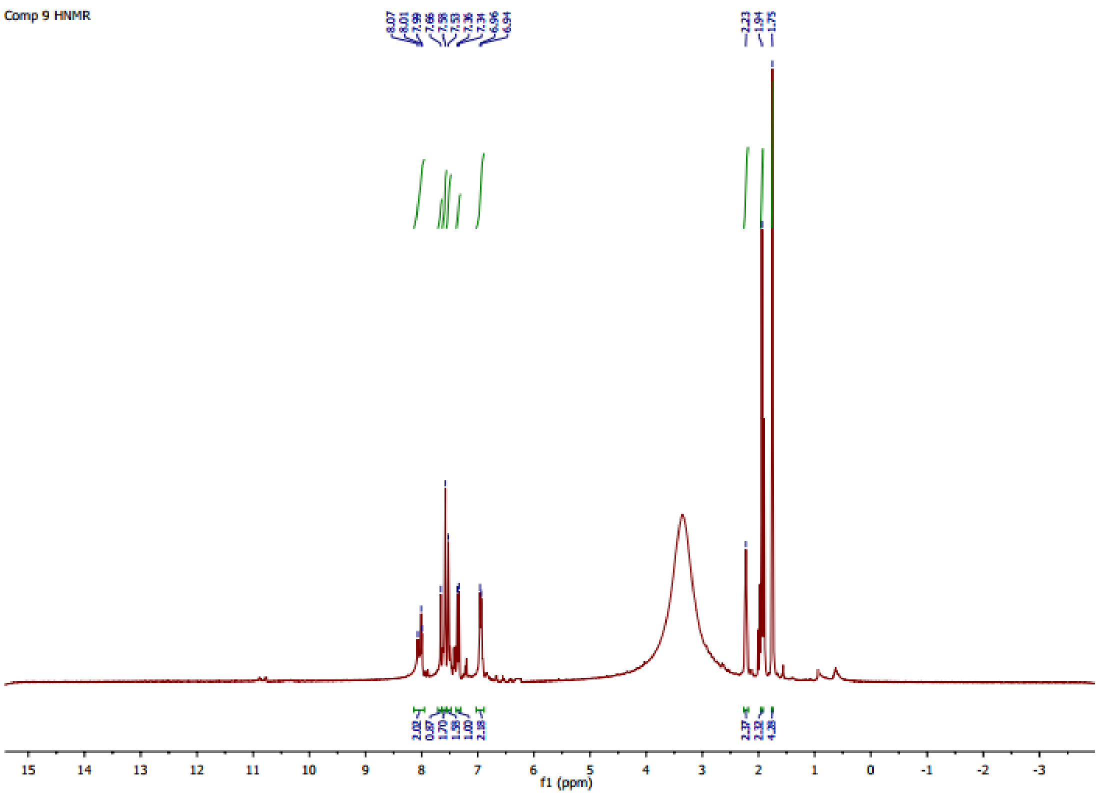

Figure S15. ^{1}H-NMR Spectrum of compound **9**

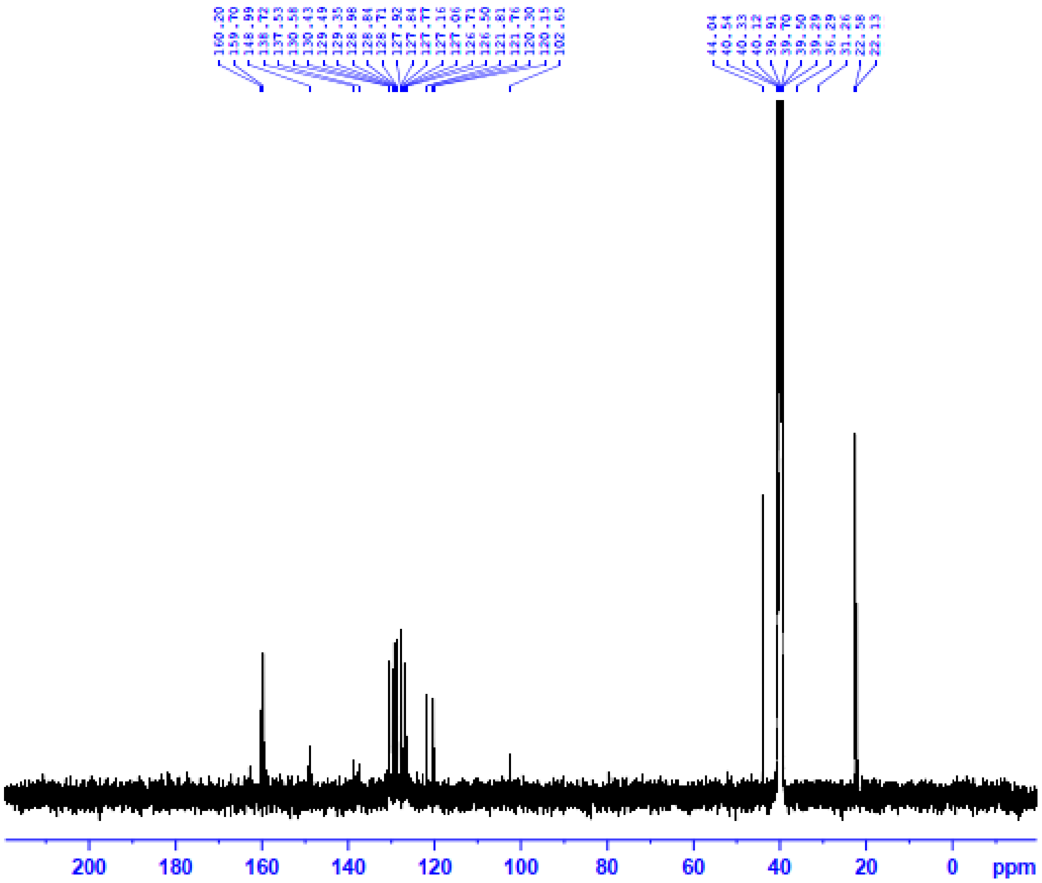

Figure S16. ^{13}C-NMR Spectrum of compound **9**
